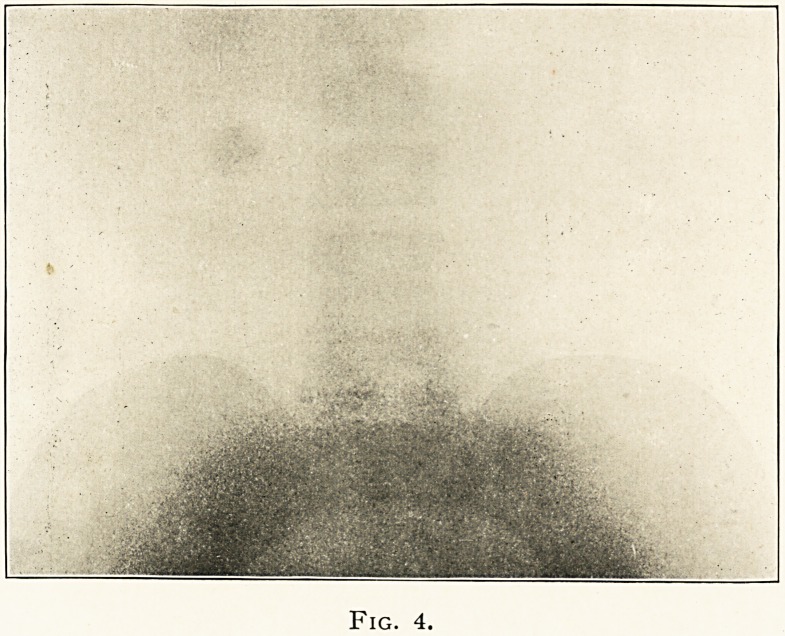# The Use of X-Rays in the Diagnosis of Renal Calculi
^1^Read before the Bristol Medico-Chirurgical Society, Nov. 13th 1901.


**Published:** 1902-03

**Authors:** James Taylor

**Affiliations:** Skiagraphist to the Bristol Royal Infirmary


### THE USE OF X-RAYS IN THE DIAGNOSIS OF RENAL CALCULI.[1]

BY

James Taylor, M.R.C.S.,
*Skiagraphist to the Bristol Royal Infirmary.*

In the early days of skiagraphy it was considered somewhat of an achievement to get a shadow of a stone in the kidney, and it was only after a prolonged exposure of half an hour or more to the rays that any results were obtained; but now, thanks to

[1] Read before the Bristol Medico-Chirurgical Society, Nov. 13th, 1901.

improvements in the apparatus employed, it is a much easier matter: the exposure is now only a question of a few minutes, and in special cases skiagrams of the kidney region have been taken in a few seconds. Even with improved means, it is not always easy to get a satisfactory skiagram. When we take into consideration that muscle is not very pervious to the rays, and fat still less so; also, when we remember that, in addition to the rays having to penetrate the muscle and fat of the abdominal walls and the omentum, we have to reckon with the contents of the intestines which are in constant movement during respiration, it is not to be wondered at that the resulting shadow of a foreign body (like a stone in the kidney) should be somewhat blurred and indistinct. In young people and women, where there is little or no muscular development, the task of taking a satisfactory skiagram of the region of the kidney is a fairly easy one; but when it comes to having to deal with men with well-developed abdominal muscles, and a large deposit of fat as well, it is a very different matter, and even with much more prolonged exposure to the rays one cannot always get a good result. Mr. J. Hutchinson, jun.,[1] draws attention to the fact that in very fat persons the use of the X-rays is valueless in the detection of renal calculi. Moreover, the calculi themselves are a source of difficulty, for some are more pervious to the rays than others, as was shown by Dr. Swain in his paper on the subject in the *Bristol Medico-Chirurgical Journal* of 1897.[2] This observer showed—and Mr. Harnack, in Mr. Hutchinson's paper already mentioned, bears out his conclusions—that oxalate of lime calculi were the most impervious to the rays and consequently threw the deepest shadow on the photographic plate, that phosphatic calculi were rather more pervious and threw a less dense shadow, and that uric acid calculi were still more pervious and threw a comparatively slight shadow. This being so, it stands to reason that if a too long exposure were given the rays would penetrate a uric acid stone and the presence of a calculus would be overlooked. Consequently, it is always wise to take more than one skiagram, with different lengths of exposure, in a suspected case of calculous disease.

[1] *Brit. M. J.*, 1901, ii. 1130. [2] Vol. xv. 1.

The value of skiagraphy in demonstrating the presence of a stone or stones in the kidney, and in clearing up a doubtful diagnosis, is well shown in the following cases, which have occurred at the Bristol Royal Infirmary during the past year, and which I am enabled to publish through the courtesy of the various members of the surgical staff.

**Case 1.**—E. F., aged 30, admitted under the care of Mr. Bush, November 20th, 1900, complaining of pain in the right side. She had an illness six years ago which confined her to bed for two weeks, during which illness bad pain in the back was a prominent symptom. She has had four or five attacks of pain in the back since, and has passed "matter" in the urine, but has never passed blood. She has had persistent pain in the right side for the last four months. There has been an occasional rise of temperature morning and evening. On admission an enlarged movable right kidney could be plainly felt. There was some pus in the urine but no blood, and the only microscopical deposits were a few crystals of oxalate of lime. Tubercle bacilli were carefully searched for without result. The lungs were normal. A skiagram taken on November 26th (Fig. 1) showed a large calculus in the lower part of the right kidney. At the operation on November 27th the kidney was found enlarged and cystic, and on incising it thin pus and urine escaped. A large saddle-shaped stone was found and removed from the lower part of the pelvis of the kidney. The patient was discharged cured on December 24th.

There was some doubt in this case as to whether the symptoms were not due to tubercular disease of the kidney, and not to calculus, but the use of the X-rays made certain the presence of a stone.

**Case 2.**—A. B., aged 21, was admitted under the care of Mr. Prichard, February 23rd, 1901. In this case there was a history of the usual symptoms of stone in the kidney, the man having suffered with renal colic, hæmaturia, and gravel for nearly two years. The skiagram (Fig. 2) confirmed the diagnosis, and showed the stone to be roundish in shape and situated in the upper part of the right kidney. The stone, which chiefly consisted of calcium oxalate, was successfully removed on March 18th, and the patient left the Infirmary well on April 25th.

**Case 3.**—M. A. J., a woman aged 30, was admitted under the care of Mr. Carwardine, April 20th, 1901. She had been operated on eight years before by the late Mr. Greig Smith, who removed three large and several small stones from the right kidney. She kept well for two years after this. In 1899 she became pregnant, and after the pregnancy a swelling formed in

Fig. 1.

Fig. 2.

FIG. 3.

FIG. 4.

the site of the wound where the drainage tube had been inserted at the time of the operation. This swelling was "lanced," and since then the wound has never healed, but has kept discharging. On examination after admission, a globular mass, about the size of a cricket-ball, could be felt in the right loin, and over this swelling there was a sinus, which admitted a probe for a distance of three inches. The left kidney could also be felt. The urine contained pus. A skiagram (Fig. 3) was taken, which showed three calculi in the right kidney, two—or what looked like two—in the middle of the loin, and one nearer to the spine. At the operation, what appeared to be two calculi in the skiagram proved to be the two processes of a single rectangular calculus. This stone was removed after burrowing into the scar tissue, the line of operation being determined by the skiagram. The second calculus was found deep down towards the spine, and was only discovered after a careful search had been made for it by boring with the finger in the precise direction indicated in the skiagram. The calculi after removal weighed 152 grains, and were composed of phosphate and a little carbonate of lime. The patient was discharged to the Convalescent Home on May 30th.

The invaluable aid afforded by the X-rays is well exemplified in this case, for it is almost certain that in the days before the introduction of skiagraphy the second stone would have been overlooked, and the operation would have been a failure. The surgeon, when he had removed, and had carefully examined the space in which such a large stone lay and found no other foreign body, would naturally think that he had done all that was necessary, and the operation would have failed to give relief. Without the skiagram the second stone would have been missed; without its aid to direct him, it would have been distinctly dangerous for the surgeon to have searched for it so close to the vena cava.

**Case 4.**—A. S., aged 31, was admitted under Mr. Munro Smith, May 29th, 1901, suffering from a typical attack of renal colic. He gave a history of an illness at 9 years of age, and from that time to the present he has had frequent attacks of pain in the right side, and has often passed blood in the urine. A skiagram was taken, which showed a calculous mass in the right kidney, the kidney being apparently outlined by it. At the operation on June 5th a huge stone was removed, which weighed 462 grains, consisting of phosphate of lime. During the next few days after the operation he passed a large number of small calculi (some of which were composed of calcium oxalate) *per urethram*, but with that exception made an uneventful recovery, and was discharged well on July 1st.

This skiagram is not reproduced here, as although the shadow of the stone was perfectly distinct on the photographic plate, yet it was not of sufficient density to produce a print which would show the calculi.

**Case 5.**—W. Y., aged 13, was admitted to Mr. Bush's ward, October 8th, 1901. He was in the Infirmary two years previously suffering from hæmaturia, on which occasion he was sounded for stone with a negative result. After a short stay in the Infirmary the hæmaturia ceased, and he went out apparently well. Since this date he has had occasional attacks of pain in his side, but no hæmaturia till the present attack. On admission he complained of increased frequency of micturition, but there was no dysuria. The urine was acid and contained blood. Microscopically, besides blood corpuscles, pus cells were present. On October 11th a sound was passed into his bladder, but no stone could be detected. A skiagram was taken on October 18th (Fig. 4), which distinctly showed a rounded stone in the upper part of the right kidney. Nephrolithotomy was performed on October 22nd, and the boy was discharged well on November 11th.

In this case, where the symptoms were somewhat doubtful, the skiagram was of special value, for it not only demonstrated the presence of calculous disease in the kidney, but also showed with certainty which kidney was affected and the part of the organ in which the stone was situated. The latter point was of assistance at the operation, because, when the kidney was exposed, no stone could be felt on manipulation, and the skiagram served as a guide to the direction in which to pass the exploring needle.

To sum up, the use of the X-rays in these five cases confirmed the diagnosis of renal calculus arrived at by ordinary clinical methods in Cases 2 and 4; demonstrated the presence of a stone in a possibly tuberculous kidney in Case 1; showed the disease to be renal, and which kidney was affected, in Case 5; and in Case 3, it not only showed the stones to be multiple, but proved of invaluable assistance to the surgeon in operating.

## Figures and Tables

**Fig. 1. f1:**
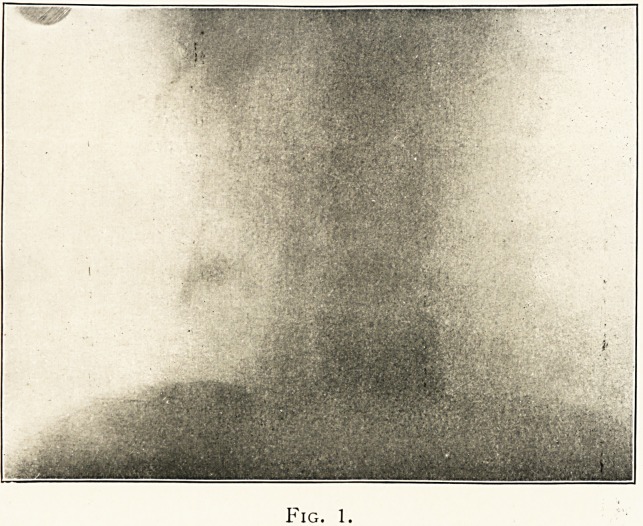


**Fig. 2. f2:**
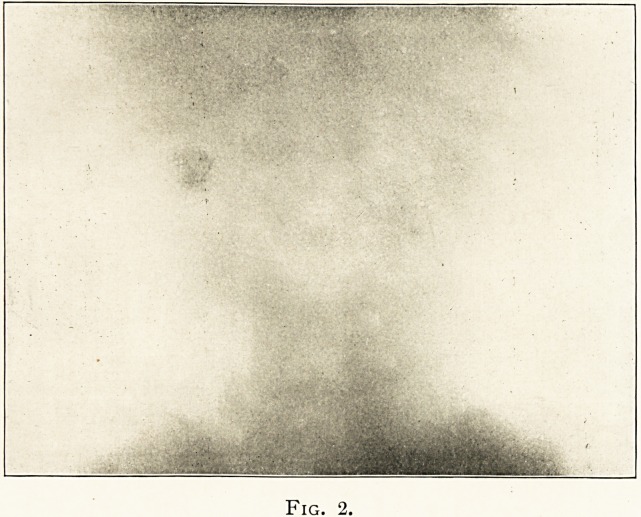


**Fig. 3. f3:**
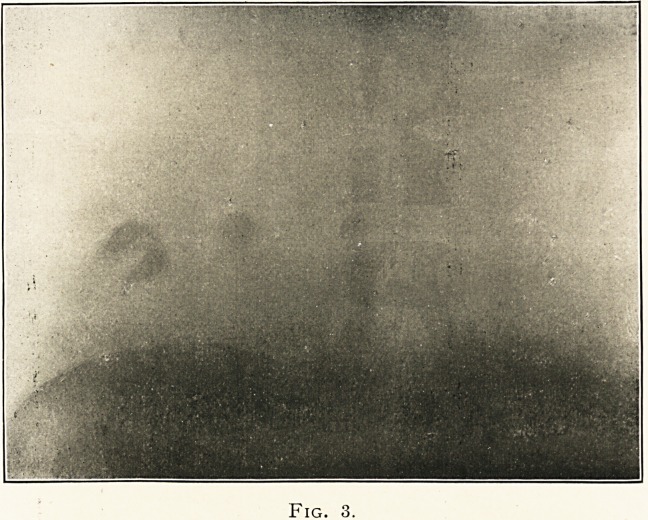


**Fig. 4. f4:**